# Attachment Style During a Partner’s Deployment with the United States Navy: Associations with Relational Satisfaction, Stress, and Changes over Time

**DOI:** 10.3390/ijerph22071056

**Published:** 2025-06-30

**Authors:** Alaina M. Hansom, Laura K. Guerrero

**Affiliations:** 1 The Henry M. Jackson Foundation, Inc., In Support of the Consortium for Health and Military Performance, a DoD Center of Excellence, Uniformed Services University, Bethesda, MD 20814, USA; alaina.hansom.ctr@usuhs.edu; 2 The Hugh Downs School of Human Communication, Arizona State University, Tempe, AZ 85287, USA

**Keywords:** attachment styles, affection, affectionate writing, expressive writing, military deployment, relational satisfaction, stress

## Abstract

Separation from a loved one can trigger the attachment system and cause stress, especially for those with insecure attachment styles. The present study investigates how attachment style relates to the degree of stress and relational satisfaction individuals experience during one such situation—that of being separated from a partner who is on military deployment. Findings from a questionnaire distributed while participants’ partners were deployed show that secure attachment is positively related to relational satisfaction, whereas preoccupied and fearful attachment are negatively related to satisfaction. In addition, having a preoccupied or fearful attachment style is positively associated with experiencing deployment stress, whereas having a secure or dismissive style is negatively related to experiencing deployment stress. This study also addressed whether attachment style might change over the course of deployment and if affectionate writing would lead people to report becoming less insecure. Results from a quasi-experiment using a pre-test–post-test design showed that those who engaged in affectionate writing (versus innocuous writing or no writing) reported less preoccupied attachment over time. Both affectionate and innocuous writing led to less fearful attachment over time in comparison to a no-writing condition. Thus, communicating via writing may be an important tool for reducing attachment insecurity during military deployments.

## 1. Introduction

Partners of military service members face many challenges, including being at increased risk of developing insecure attachments [1]. Military deployments can disrupt the attachment system, producing feelings of stress and causing partners to detach from one another or feel insecure [2]. The attachment system is activated when individuals are distressed, leading them to seek proximity to caregivers (as children) and later to loved ones to feel safe and secure. Being apart from a romantic partner can trigger this system. Separation also prevents partners from engaging in some of the proximity-seeking behaviors that help them maintain a secure and satisfying relationship. Indeed, some of the stress couples experience during deployments is related to trying to maintain their relationship while apart [3]. To examine attachment during military deployments, the present study surveys partners of service members (the vast majority of whom were living on base while their partner was away) to determine how their attachment style associates with relational satisfaction and stress during deployments. In addition, an experiment is conducted to determine if engaging in affectionate communication via expressive writing produces changes in attachment style for the at-home partners of service members.

### 1.1. Military Couples as a Context for Studying Separation

Military deployments provide a rich space for studying attachment and relationship dynamics given the stress and uncertainty that can occur when partners are physically separated from each other [4]. Military couples commonly experience many stressors in their relationships, particularly during deployment. Couples must cope without each other on a daily basis, while sometimes worrying about the potential dangers of military service. Specific challenges include marital problems [5], divorce [6], distress [7], decreased troop morale [8], a lack of communication [9], and decreased relational satisfaction [10]. Some or all of these challenges are experienced by the approximately 1.3 million people actively serving in the United States (U.S.) military [11], of which about 15% are deployed overseas each year [12].

U.S. Navy families may be at particular risk of experiencing high levels of stress during deployment [13], impacting the effectiveness of service members while on duty [14]. Compared to the stress experienced during non-deployed shore tours, stress is highest during deployments [15,16]. This could be because U.S. Navy deployments differ from deployments associated with other branches of the military. For example, until recently, during deployments on U.S. Navy ships, such as aircraft carriers and submarines, military personnel did not have wireless internet or reliable phone service [17]. In 2023, the Navy started testing the roll-out of Wi-Fi on one ship [18], but communication between service members on a ship and their families at home remains limited on other ships. Furthermore, unlike other branches of the military, the U.S. Navy has regularly scheduled deployments [19] regardless of current conflict.

Partners of deployed service members are at heightened risk for stress because of the schedule of deployments that they endure. Stressors include worrying about the partner’s safety, missing the partner and feeling lonely, having to deal with responsibilities alone, and waiting for communication from the partner [20]. Deployments are a stressful time for both service members and their spouses, as the absence of a loved one changes the family environment [21]. The Fleet and Family Support Center outlines the emotional rollercoaster that couples and families go through before, during, and after such deployments [22]. Thus, a service member’s deployment is likely to activate the attachment system and provide a suitable context for investigating how attachment style relates to stress, as well as relational satisfaction during periods of separation. Data collection for the current study was conducted with female spouses or significant others of deployed service members in the U.S. Navy.

### 1.2. Attachment, Stress, and Relational Satisfaction

Individuals have different styles of attachment that influence their thoughts, emotions, and communication, particularly during times of stress such as separation. Attachment styles are based on models of the self and others [23]. The model of self encompasses the degree to which people have a positive or negative image of themselves. Those with positive models of self see themselves as worthwhile and lovable. The model of others reflects the extent that others are viewed positively or negatively. When people have a positive model of others, they generally expect people to be responsive and caring rather than unresponsive and uncaring.

Based on the combinations of models of self and others, Bartholomew and Horowitz [23] proposed four adult attachment styles: secure (positive model of self, positive model of others), dismissive (positive model of self, negative model of others), preoccupied (negative model of self, positive model of others), and fearful (negative model of self, negative model of others). Each of the four attachment styles have distinct characteristics [23,24,25]. Specifically, individuals with a secure attachment style are comfortable with both autonomy and closeness; this means they value relationships but also value their independence. They tend to have satisfying relationships and deal well with stress. Individuals with a dismissive attachment style are self-confident and extremely self-sufficient; they do not fear or desire closeness and are instead so self-sufficient that they shun closeness. Thus, they should be able to handle separation well given that they are not as attached to their partners as are those with other attachment styles. Individuals with a preoccupied attachment style are overly dependent on relational partners; they need to have a relationship to feel worthwhile and worry their partners will abandon them, which can lead to relationship dissatisfaction and stress being exacerbated during separation. Finally, individuals with a fearful attachment style have usually been hurt or rejected in the past so they fear closeness; they want close relationships but have difficulty opening up to others because they fear getting hurt again, leading them to feel especially vulnerable when separated from loved ones.

As these descriptions suggest, attachment is fundamentally related to separations and reunions. Threats to attachment, such as the unavailability of an attachment figure during deployment, motivate individuals to maintain or restore proximity to their attachment figures [26,27]. Separation during military deployment may be especially challenging for insecurely attached individuals who have trust or fear of abandonment issues, namely those with preoccupied or fearful attachments. For these individuals, long separations and missed attempts to connect during deployment could worsen these issues [28], increase stress, and reduce satisfaction [29]. Securely attached individuals, in contrast, may view deployment as a temporary time apart and, therefore, remain committed to the relationship [29]. Typically, dismissive attachment is associated with relatively low relationship satisfaction. During deployment, however, dismissive individuals may welcome time to focus on themselves and exercise more autonomy in their daily lives, making it difficult to predict what the association between relational satisfaction and dismissive attachment might be during deployment.

Research also shows that both secure and insecure individuals may demonstrate some degree of ambivalence and anger post-deployment [27]. Indeed, re-entry into one another’s lives can be stressful for couples when reunited since partners need to readjust their expectations and routines [30]. However, secure individuals tend to adjust to deployment transitions easily and positively. One study, for example, found secure individuals to report lower levels of conflict and higher levels of relational satisfaction than preoccupied individuals when separated during Desert Storm [31]. Other studies have shown that both attachment avoidance and attachment anxiety are positively associated with depression and PTSD for service members and veterans [32,33]. The present study extends this research by examining whether there are associations between attachment style, stress, and relational satisfaction for partners of service members during deployments.

**H1.**  *Relational satisfaction is (a) positively associated with the extent to which at-home partners of deployed service members have a secure attachment style and negatively associated with the extent to which at-home partners of deployed service members have a (b) preoccupied or (c) fearful attachment style.*

**RQ1.**  *Is there an association between relational satisfaction and the extent to which at-home partners of deployed service members have a dismissive attachment style?*

**H2.**  *Stress is negatively associated with the extent to which at-home partners of deployed service members have a (a) secure or (b) dismissive attachment style and positively associated with the extent to which at-home partners of deployed service members have a (c) preoccupied or (d) fearful attachment style.*

### 1.3. Shifts in Attachment Style and Affectionate Writing

When people are separated from a close relational partner, they will engage in proximity-seeking behaviors to maintain close affectional bonds. During deployments, however, couples face challenges communicating with each other on a daily basis. Although they may be able to exchange some information with one another, it is difficult to show affection when physically separated. This is problematic because affectionate communication has been associated with physical and mental health, as well as relational benefits [7]. Scholars recommend increasing expressions of affection to improve physical and mental health and keep relationships healthy [34,35]. Military couples may find it difficult to follow this advice given their unique circumstances during deployment. The second part of this study investigates whether engaging in an affectionate writing task during deployment influences people’s levels of attachment (in)security.

Although attachment styles are relatively stable across relationships and time [36,37], in some studies, approximately 25–30% of adults report that their attachment style changed over time [38,39]. Attachment shifts are linked to critical events, such as divorce, death, commitment, and distance [40]. Deployment creates the type of distance that can trigger the attachment system [2], causing individuals to feel more insecure while apart from their partners. Having a secure attachment style may provide a buffer against this, but thus far no research has examined shifts in attachment style during deployment.

Given that attachment is strongly related to proximity seeking, it follows that couples who find ways to connect even when separated by distance will feel more secure than couples who do not. This is because, from an attachment theory perspective, being mentally and emotionally connected to a partner during separation helps to maintain a sense of security. Communication that promotes connection during deployment can reduce anxiety and enhance mental health [41]. Thus, it is not surprising that engaging in positive communication with loved ones is negatively related to depression and PTSD for service members [42]. When partners cannot engage in direct communication, other activities might keep them connected. For example, in one study, female partners of U.S. service members engaged in a stream-of-consciousness task that involved talking aloud about what they expected their reunion with their partner to be like [4]. These dialogues were then coded for evidence of relational savoring (i.e., anticipating positive emotions in conjunction with re-connecting to their partner). Although psychological distress and low relationship satisfaction were linked for many participants, for participants who engaged in relational savoring, they were not.

When direct communication with one’s partner is limited, expressive writing may provide another way for couples to express emotions and feel connected despite separation. Pennebaker developed the expressive writing paradigm to demonstrate that people who write about their feelings on a regular basis are better able to deal with loneliness or the aftermath of traumatic events [42,43,44]. For example, studies have shown that compared to a control group who wrote about innocuous topics such as their plans for the day, participants who wrote about their most traumatic experiences were subsequently physically and mentally healthier [45,46,47]. Similarly, those who engage in expressive writing are better able to cope with significant personal challenges or life transitions [48]. A meta-analysis showed that although the positive effects of expressive writing tend to be relatively small, they tend to persist over time [49].

Although most work on the expressive writing paradigm has focused on traumas or illness, some work has examined affectionate communication and emotional expression more generally. Specifically, Floyd and his colleagues applied Pennebaker’s expressive writing paradigm to an affectionate writing intervention. The researchers conducted a two-part experiment exploring the impact of affectionate writing on total serum cholesterol reduction over a 5-week period [50]. However, instead of having the participants write about their traumas, the researchers directed the participants to write about their positive and affectionate feelings toward three close relational partners: a close friend, a close relative, and the person whom they consider to be the closest person in their lives. A control group wrote about innocuous or non-emotional topics. As predicted, individuals in the affectionate writing group had a reduction in total cholesterol following the intervention. In a study on military couples, relational satisfaction increased when soldiers who served in Iraq and Afghanistan wrote emotional letters to their spouses [51].

Overall, it appears that expressive writing provides opportunities for reflection on romantic relationships, leading to health and relational benefits. The process of writing helps participants to connect to their emotions, create a narrative for the events in their life, and sometimes even modify how they think about events, leading to positive changes in attitudes and behaviors [52,53]. This reasoning could extend to attachment security. By engaging in expressive writing, at-home partners of deployed service members may be better able to cope with the separation, leading them to experience less feelings of insecurity in their relationships. From a theoretical standpoint, behaviors that help people to cope better with a separation may also help people to feel more secure. Indeed, expressive writing may function as an indirect proximity-seeking mechanism that helps people to feel connected and more secure during times of separation when the attachment system is likely to be activated. If this is the case, people should experience increases in security and decreases in insecure forms of attachment if engaging in expressive writing compared to engaging in no writing or an innocuous writing task. This reasoning leads to a final research question:

**RQ2.**  *Are there changes in at-home partners’ attachment styles based on whether they engage in affectionate writing versus innocuous writing or no writing?*

## 2. Materials and Methods

### 2.1. Participants

To recruit anonymous at-home partners from the target population, participants were recruited via listservs of ROTC alumni of the first author’s prior academic institutions. These institutions are large universities geographically located in the Southeast and Southwest regions of the United States with diverse alumni populations. The participants’ partners were stationed in various places around the United States. A priori power analysis was conducted using G*Power 3.1. For a repeated measures design that included a 2 (within-subjects/time) × 3 (between-subjects/condition) interaction, with an effect size for *F* set at 0.20 and power set at 0.80, a minimum of 66 subjects (22 per condition) was necessary. The authors therefore aimed for a minimum of 25 participants per cell. The final sample consisted of 80 women who were randomly assigned to one of three groups: the affectionate writing group (*n* = 25), the control/innocuous writing group (*n* = 27), or the control/no-writing group (*n* = 28). Conditions were determined at the time instructions were sent out, with the first author drawing conditions out of a hat in the order in which participants signed up. All participants completed questionnaires during an initial data collection that occurred when their partners were deployed and then a month later. Between the two data collection points, some participants were instructed to engage in writing activities, whereas some were not, as described later. The study protocol was approved by Arizona State University’s Institutional Review Board. Participants were informed that they could stop participating any time and were debriefed at the end of this study.

The mean age of participants was 30.01 years (*SD* = 6.25). Participants identified ethnically as Caucasian/white (*n* = 44, 55%), Latinx/Hispanic (*n* = 24, 30%), African American (*n* = 9, 11.30%), and Native American (*n* = 2, 2.5%), with one participant not disclosing her ethnicity. The reported highest level of education was a high school degree (*n* = 36, 45%), a bachelor’s degree (*n* = 32, 40%), some college (*n* = 7, 8.80%), professional school (*n* = 3, 3.8%), and a master’s degree (*n* = 2, 2.5%). Within each condition, 11 to 15 participants had college degrees, with the remaining participants having graduated from high school or completed some college. The average length of their romantic relationship was 6.69 years (*SD* = 4.48), with most participants being married (*n* = 72, 90%) and the rest dating (*n* = 5, 6.30%) or engaged (*n* = 3, 3.80%). Whereas most participants did not have children (*n* = 45, 56.30%), the rest reported having children (*n* = 22, 27.50% had one child; *n* = 11, 13.80% had two children; *n* = 1, 1.30% had three children; *n* = 1, 1.30% had four children). Most participants lived on base (*n* = 77, 87.50%). Almost 60 percent of the participants’ romantic partners were enlisted (*n* = 47, 58.80%), and the rest were commissioned officers (*n* = 33, 41.30%) in the U.S. Navy. The average number years their romantic partner had been in the U.S. Navy was 4.35 years (*SD* = 1.79), and the average number of previous deployments the couple had endured was 3.36 (*SD* = 1.67). Finally, the average length of the current military deployment was 14.83 weeks (*SD* = 5.62). By the conclusion of this study, half of the participants stated that their partner had recently returned home from deployment (*n* = 40, 50%), and the other half reported their partner was still currently deployed (*n* = 40, 50%).

### 2.2. Pre-Screening Procedure

To determine their eligibility for this study, prospective participants completed pre-screening checks ensuring that they were women of at least 18 years of age; they were able to speak, read, and write English; and their romantic partners were service members in the U.S. Navy who were currently deployed. Although the preliminary goal was for the military partners to be deployed during the duration of the experiment, as noted above, by the conclusion of this study, half of the participants reported that their partner had recently returned home from deployment. The choice to include only women reflects the fact that there are more than three times as many men than women active in the United States military [11] and 90% of military spouses are female [54].

### 2.3. Experimental Procedures

After consenting to participate in this study, qualified participants completed a pre-test questionnaire to collect baseline (Time 1) data on stress, relational satisfaction, and attachment style, as well as demographics. After completing the pre-test questionnaire, the participants were randomly assigned to one of three conditions—the affectionate writing (experimental) condition, the innocuous topics writing (control) condition, or the no-writing (control) condition—as described above.

Participants in both writing conditions (i.e., affectionate and innocuous) were instructed to engage in a 20-min writing activity once a week on Sunday for three consecutive weeks. The participants were initially contacted via email at 10am (CDT). Any participants who did not complete their writing prompts by 10am on Monday were sent an email reminder. For each writing activity, participants were offered one of three topics associated with their condition to write about, with the order of the topics randomly assigned. Participants were specifically told not to focus on spelling, punctuation, or grammar, but rather to focus on the content of their response to the writing prompt. Further, participants in the experimental group were told to write *to* their romantic partner rather than about them [39], although the responses were not shared with their romantic partners (only the first author had access to the responses). Participants were instructed to write for the entire 20-min duration of the writing activity; the online questionnaire had a 20-min countdown timer on the writing prompt page and participants could not click to the next page until the timer ran out. At the end of this study, all participants completed the post-test questionnaire, including the attachment style measures.

In random order, each participant in the affectionate writing experimental group was instructed to respond to each of the following prompts, similar to past studies [50]:

1. Think about the little things you miss about your romantic partner. Write a letter to your romantic partner describing the little things you miss about him. You may also write about your partner’s positive qualities (or the qualities that make you miss him).
2. Think about how much your romantic partner means to you. Write a letter to your romantic partner describing your loving and caring feelings for him. You may also write about the reasons why you love your partner.
3. Think about how much you appreciate your romantic partner. Write a letter to your romantic partner describing your appreciative feelings for him or her. You may also write about the things that your partner does that makes you appreciate him.

In random order, each participant in the innocuous writing control group was instructed to respond to each of the following prompts modeled after previous research [50]:

1. Think about the television programs that you watched this past week. Give a detailed description of the television programs that you watched this past week.
2. Think about the home you currently live in. Give a detailed description of your current residence.
3. Think about your current job or the last job that you held. Give a detailed description of how you spent your time at work and the overall work environment in which you worked.

### 2.4. Compensation Procedure

Participants received compensation routinely throughout this study. Specifically, they received USD 5 for completing the pre-test questionnaire, USD 10 per writing session (USD 30 in total for the three writing sessions), and USD 5 for completing the post-test questionnaire. The periodic payment encouraged participants to continue their participation for the duration of this study, but it did not force them to complete the study in full to receive partial compensation. In total, participants could receive up to USD 40 for completing all portions of the three-week long experiment.

### 2.5. Materials

For the current study, stress and satisfaction were measured within a pre-test that the participants completed while their partners were deployed. Stress was measured by averaging items from Cohen et al.’s Perceived Stress Scale [55]. Participants assessed their agreement with the items on a 5-point Likert scale (1 = never; 5 = very often). Wording on the scale was altered to represent deployment as a time frame rather than the previous month (which was the time frame for the original items). Because the 10 items in the original scale were modified to refer to deployment, an exploratory factor analysis using the maximum likelihood method and direct oblimin rotation was used to check the unidimensionality of the revised scale. Initial results revealed two factors, with three of the items that had been re-coded loading on a second factor. After these items were dropped, the seven remaining items loaded on a single factor, with factor loadings ranging from 0.52 to 0.95. These items (α = 0.83; *M* = 2.87, *SD* = 0.45) included “Since your partner was deployed, how often have you been upset because of something that happened unexpectedly” and “Since your partner was deployed, how often have you felt difficulties were piling up so high that you could not overcome them?” Relationship satisfaction was measured using the five positively worded items from Hendrick’s 7-item Relationship Assessment Scale [56]. Participants assessed their agreement with the items on a 5-point Likert scale. The five items (α = 0.85; *M*= 5.43, *SD*= 0.62) included “How well does your partner meet your needs?” (1 = not very well; 5 = very well) and “How good is your relationship, compared to most?” (1 = not good at all; 5 = very good).

Attachment style was measure twice—once along with the other pre-test measures, and then again at the conclusion of the experiment. Attachment style was assessed using an adaptation of Guerrero et al.’s 25-item attachment style assessment [25]. Participants assessed their agreement with the items on a 7-point Likert-type scale (1 = strongly disagree; 7 = strongly agree) and were scored on the four adult attachment styles: secure (time 1 α = 0.77, *M* = 5.66, *SD* = 0.71; time 2 α = 0.78, *M* = 5.68, *SD* = 0.76), preoccupied (time 1 α =.73, *M* = 4.84, *SD* = 1.07; time 2 α = 0.76, *M* = 4.54, *SD* = 1.15), dismissive (time 1 α = 0.77, *M* = 5.34, *SD* = 1.30; time 2 α = 0.72, *M* = 5.35, *SD* = 0.97), and fearful (time 1 α = 0.75, *M* = 4.68, *SD* = 1.55; time 2 α = 0.82, *M* = 4.17, *SD* = 1.55). Sample items included “I find it relatively easy to get close to people” for secure, “I wonder how I would cope without someone to love me” for preoccupied, “I feel smothered when a relationship takes too much time away from my personal pursuits” for dismissive, and “I avoid getting close to others so I won’t get hurt” for fearful.

### 2.6. Data Analysis

Prior to analyzing the data, we tested for possible control variables, including the number of deployments the romantic couples had previously endured, the length of the current deployment, and the number of years the partner had been in the military, to determine if any of these variables were significantly associated with relational satisfaction, stress, or attachment style. Bivariate correlations revealed that having endured more deployments in the past was negatively associated with stress during the current deployment (*r*(78) = −0.35, *p* < 0.01). Thus, the number of previous deployments served as a control variable for the analysis that included stress. No other significant correlations with potential control variables emerged.

We also conducted ANOVAs (see Table 1) to determine whether those assigned to the three conditions varied in terms of age, relationship length, length of the current deployment, number of deployments they had experienced while together, number of years the service member had been in the military, and attachment style at Time 1. The results showed that none of these variables differed significantly based on condition. Finally, *t*-tests were conducted to determine if there were differences in variables measured at Time 2 based on whether the U.S. Navy service member had recently returned home or was still deployed. As shown in Table 2, there were no significant differences.

To test the predicted associations among attachment style, relational satisfaction, and stress, we conducted regression analyses among these variables, as reported at Time 1. Thus, these analyses represent how these variables were related to each other during an early-to-middle phase of deployment. To test whether there were changes in attachment style based on participation in the affectionate writing exercise, a series of contrasts were conducted within 2 (time; as a repeated factor) by 3 (condition; as a between factor) ANOVAs and probed by looking at simple main effects.

## 3. Results

### 3.1. Associations Among Attachment and Relational Satisfaction

H1 examined how the attachment styles of the at-home partners of deployed service members were associated with their relational satisfaction while they were apart. Specifically, the hypothesis predicted that secure attachment (H1a) is positively associated with relational satisfaction, whereas preoccupied (H1b) and fearful (H1c) attachment are negatively associated with relational satisfaction. A research question addressed the possible association between dismissive attachment and relational satisfaction. The regression analysis was significant (*F*(4,74)= 25.63, *p* < 0.001, *R*^2^ = 0.58). As shown in Table 3, all parts of the hypothesis were supported. In response to RQ1, the association between dismissive attachment and relational satisfaction was non-significant.

### 3.2. Associations Among Attachment and Deployment Stress

The next hypothesis examined associations among the attachment styles of the at-home partners of deployed service members and their reported levels of stress during deployment. These hypotheses predicted that secure (H2a) and dismissive (H2b) attachment styles would be negatively associated with stress, whereas having a preoccupied (H2c) or fearful (H2d) attachment style would be positively related to stress. The number of previous deployments was entered in the regression analysis as a control variable in the first step, followed by the attachment style variables in the second step, with deployment stress as the criterion variable. The model was significant after the first step, i.e., *F*(1,79) = 9.99, *p* < 0.01, *R*^2^ = 0.12, with previous number of deployments negatively associated with deployment stress (*b* = −0.09, SE = 0.03, β= −0.34, *t* = −3.16, *p* < 01). The model improved significantly when the attachment style variables were added in the second step (*F*(5,73) = 11.01, *p* < 0.01, *R*^2^ = 0.39, *F*_change_(4) = 10.08, *p* < 0.01). As shown in Table 3, the control variable (number of deployments) lost significance after the attachment variables were entered in the model, but all parts of H2 were supported.

### 3.3. Changes in Attachment Styles as a Function of Expressive Writing

The second research question asked if attachment styles change across the deployment cycle based on the writing condition. For secure attachment, contrasts showed no significant effects for time, i.e., *t*(77) = 0.64, *p* > 0.05; condition, i.e., *t*(77) = 0.08, *p* > 0.05; or the time-by-condition interaction, i.e., *t*(77) = 0.71, *p* > 0.05. Thus, secure attachment remained relatively consistent from Time 1 to Time 2 during the deployment cycle.

For preoccupied attachment, there were significant effects for time, i.e., *t*(77) = 3.98, *p* < 0.001, η^2^ = 0.17, and time-by-condition, i.e., *t*(77) = 1.81, *p* < 0.05, η^2^ = 0.04, but not condition, i.e., *t*(77) = 1.00, *p* > 0.05. Paired samples *t*-tests were conducted to examine the simple effects for time across conditions. The results indicated a significant decrease in preoccupied attachment for individuals in the affectionate writing condition from time 1 (*M* = 4.77, *SD* = 0.99) to time 2 (*M* = 4.27, *SD* = 1.14), *t*(24) = 4.01, *p* < 0.001, partial η^2^ = 0.40). Individuals in the two control conditions did not show a significant change from pre- to post-test. Thus, people in the affectionate writing condition were the only group that experienced a drop in preoccupied attachment over the deployment cycle.

For fearful attachment, contrasts revealed a significant effect for time, i.e., *t*(77) = 4.54, *p* < 0.001, η^2^ = 0.22, but not for condition, i.e., *t*(77) = 0.73, *p* > 0.05, or time-by-condition, i.e., *t*(77) = 0.79, *p* > 0.05. The simple effects for time showed that for those in the affectionate writing condition, there was a significant decrease in fearful attachment from the pre-test (*M* = 4.67, *SD* = 1.21) to the post-test (*M* = 4.03, *SD* = 1.63, *t*(22) = 2.63, *p* < 0.05, partial η^2^ = 0.24). There was also a significant decrease from the pre-test (*M* = 4.90, *SD* = 1.47) to the post-test (*M* = 4.38, *SD* = 1.66) in fearful attachment for those in the innocuous writing condition (*t*(26) = 3.63, *p* < 0.01, partial η^2^ = 0.34), but not in the no-writing control condition. Thus, both affectionate and innocuous writing led to decreases in fearful attachment.

For dismissive attachment, the contrasts showed no significant effects for time, i.e., *t*(77) = 0.17, *p* > 0.05; condition, i.e., *t*(77) = 0.1.57, *p* > 0.05; or the time-by-condition interaction, i.e., *t*(77) = 0.11, *p* > 0.05. Therefore, dismissiveness did not change significantly over time or by condition.

## 4. Discussion

The primary goals of this study were to investigate how attachment style is associated with stress and relational satisfaction during military deployments and how attachment styles might change during this critical juncture depending on whether at-home partners engage in an affectionate writing exercise. Data showed that during deployment, people who score high in secure attachment and low in both preoccupied and fearful attachment are most likely to be highly satisfied in their relationships. Those with high levels of preoccupied and fearful attachment are most likely to experience deployment stress, whereas those with high levels of secure and dismissive attachment are less likely to experience such stress. The results also showed individuals reported less preoccupied and fearful attachment over time if they were in the affectionate writing condition.

### 4.1. Attachment Style Correlations with Relational Satisfaction

It was expected that the attachment styles of the at-home partners of deployed service members would associate with their relational satisfaction. Specifically, having a more secure attachment style was predicted to associate with relational satisfaction, whereas having a more preoccupied or fearful style was expected to associate with relatively low levels of relational satisfaction during deployment. These predictions were fully supported and are consistent with speculation that military deployments are more difficult for insecurely attached individuals, in particular those who are anxious, than securely attached individuals [28].

The results of the current study are congruent with previous work on attachment style and military deployment. Individuals with insecure attachment styles, in this case preoccupied and fearful attachment styles, reported relatively low levels of relational satisfaction during military deployment, similar to findings from other studies [27,28]. It makes sense that individuals with abandonment (i.e., preoccupied) and rejection (i.e., fearful) issues would be less satisfied in their relationships during military deployments because their partner is away from them [25]. Further, individuals who are comfortable with both autonomy and closeness (i.e., secure) reported high levels of relational satisfaction during military deployment. Research repeatedly shows that being secure is associated with positive relational characteristics [25,57,58,59].

### 4.2. Attachment Style Correlations with Deployment Stress

Furthermore, it was expected that the attachment styles of the at-home partners of deployed service members would associate with their stress levels. Specifically, the degree to which someone possesses a secure or dismissive style was hypothesized to be inversely related to stress given their comfort with autonomy, whereas the degree to which someone has a preoccupied or fearful attachment style was predicted to be directly related to stress due to their anxiety and fear of abandonment. These predictions were supported. Both preoccupied and fearful attachment were positively related to deployment stress, which fits within the broader literature showing that individuals with insecure, especially anxious, attachments are likely to report high levels of stress [60]. Individuals with attachment anxiety (i.e., preoccupied and fearful) tend to react to threats to their attachment, report high levels of stress, and ruminate over the event [61]. They also report higher levels of stress than dismissive and secure individuals [62,63], which is consistent with this study’s finding that preoccupied and fearful attachment were positively associated with stress, whereas secure and dismissive attachment were negatively associated.

At-home partners of deployed service members who are secure may not feel much stress during deployments and instead cope well with the separation. They value both connection and autonomy, so being apart from their partner may not be overly stressful. It is noteworthy that past studies have focused on how attachment anxiety and attachment avoidance are positively related to stress during military deployment [32,33] without measuring secure attachment directly. Like secure individuals, dismissive individuals value autonomy; however, dismissive individuals are often autonomous to the point of being counter-dependent and refusing to rely on others. Thus, it is not surprising that having a dismissive attachment style would be negatively associated with stress during deployments. When at-home partners of deployed service members have a dismissive attachment style, separation may not disrupt their daily activities much and may even give them the alone time and independence that they crave, making stress unlikely.

### 4.3. Changes in Attachment Style

Importantly, there were some changes in attachment style during the deployment cycle based on the condition in which at-home partners were randomly placed. Specifically, scores on preoccupied and fearful attachment decreased from the pre- to post-test for those in the affectionate writing group. Scores on fearful attachment also decreased for those in the innocuous writing condition. There were no significant changes in attachment scores for individuals in the no-writing condition, suggesting that writing made the difference.

It appears that affectionate writing reduced preoccupied and fearful attachment from earlier to later in the deployment cycle. Individuals high in preoccupied and fearful attachment are afraid of abandonment and rejection, so writing about positive emotions may be helpful and perhaps reassuring for these individuals. Specifically, recognizing positive feelings during negative life experiences, such as deployments, is an effective way for individuals to cope [64]. Perhaps by writing about positive feelings, such as the affection that they feel toward their deployed romantic partner, preoccupied and fearful individuals can become less insecure in their relationships at a time when the attachment system is normally triggered by separation. Further, although research indicates that adult attachment styles tend to be relatively stable across relationships and time [24,37], there are reasons why attachment styles may change, including critical events [23] like deployment. Writing affectionate words during military deployment could be a critical event positively impacting attachment styles, as preoccupied and fearful individuals became less insecure after engaging in affectionate writing.

It also appears that writing about innocuous topics helped to decrease fearful attachment. It is possible that writing about innocuous topics did not pose any additional threats to fearful individuals who avoid intimacy and have difficulty disclosing emotions. For these individuals, the act of writing for 20 min once a week for three weeks may have been helpful simply because of the healing power of writing [42] and creating a cohesive narrative [65] about innocuous topics, such as careers or TV shows. Such a task may also help individuals to keep their minds off any stress or sadness about being separated from their partner.

### 4.4. Implications for Theory

Theoretically, the current study adds to the considerable literature on attachment by showing that during a critical event, such as deployment, attachment styles associate with relational satisfaction and stress in expected ways. Testing these relationships during deployment is important since this is a time when the attachment system should be activated. Perhaps even more importantly, this study adds to the debate about how stable versus malleable attachment styles are. Critical events are a time when attachment styles can move toward more or less security. At the beginning of this study, at-home partners were dealing with their loved one’s departure and the ongoing separation that they knew they would have to endure. This likely activated the attachment system, making any anxious tendencies related to preoccupied or fearful attachment salient. However, those who engaged in the affectionate writing exercises became somewhat less preoccupied and fearful over time, showing that attachment can indeed fluctuate. Importantly, attachment styles did not change for everyone. Only those in the affectionate writing condition showed a decrease in preoccupied attachment, and only those in one of the writing conditions (innocuous or affectionate) showed a decrease in fearful attachment. It is likely that other forms of communication during critical events can also make a difference. Determining those forms of communication is an important task for future research and may help to determine how proximity-seeking behavior can be used to keep attachment bonds healthy during periods of separation.

### 4.5. Implications for Practice

Scholars have also noted that secure attachments and strong relationships can act as a buffer against stress for military families facing challenges such as deployment [66]. This study suggests that it is important to identify partners of service members who have preoccupied or fearful attachment styles since they may be at more risk for experiencing stress and relational dissatisfaction, especially during times when their partner is deployed. Giving preoccupied and fearful individuals resources that help them cope during separation may be of the utmost importance for teaching them how to successfully navigate this challenging time. This study also demonstrates that attachment security is associated with relational satisfaction during deployment. Thus, programs that increase attachment security may be helpful for all couples, including military couples.

This study also offers practitioners an empirically supported intervention to execute with couples to help them feel less preoccupied or fearful during their partner’s deployment. Specifically, directing service members’ significant others to write affectionately could decrease the fear of abandonment that characterizes preoccupied and fearful attachment and that is often triggered upon separation from an attachment figure. Affectionate writing could be integrated into family readiness group (FRG) programming. FRGs aim to support the unique military lifestyle, particularly by helping families to prepare for deployment and by providing social support to families during deployments [67], and they are viewed as important groups to promote family readiness [68]. FRGs could educate members on the positive outcomes of affectionate writing, as well as provide resources to members interested in affectionate writing, such as journals or quiet spaces for writing. Such an approach is consistent with recommendations to use emotionfocused therapies that involve expressing positive feelings to promote strong attachment bonds during military deployments [66]. Practitioners may use expressive writing along with tasks encouraging relational savoring [4] as part of emotion-based therapies [66].

The current evidence on the effectiveness of resilience programming for military families is mixed [68,69]. Few evidence-based interventions have been developed or tested for military families [70]; in fact, a review of programs developed for military couples during deployment reveals that only two programs are available, both of which have unclear findings [71]. Practically, the results of this study could introduce theoretical backing to programs, assist in testing the effects of already-established programs, and provide a theoretical foundation for future programs. Research suggests that expressive writing has small long-term effects on anxiety, depression, and other mental health indicators, especially when there are short intervals between the writing sessions [49]. This may extend to concepts like fear of abandonment and attachment anxiety. Thus, one recommendation is to implement affectionate writing as a frequent part of a regular routine during deployments. Research should examine whether decreases in attachment insecurity due to affectionate writing during deployment persist over time and whether integrating affectionate writing into existing programs will enhance those programs’ effectiveness.

## 5. Limitations and Conclusions

One strength of the current study is the focus on the under-studied population of military partners [72], especially during deployment. However, there are several limitations regarding the sample. The findings cannot be generalized to all at-home partners of U.S. Navy service members since this study did not include male civilian partners or LGBTQ+ relationships. Future research should focus on these relationships so their experiences can be represented in the empirical literature [73]. The findings also cannot be generalized to other branches of the U.S. military or to military forces around the world due to cultural differences. Over 87% of participants were also living primarily on a military base, so findings are most generalizable to this group. In future work, samples including more partners living off base would allow for a meaningful comparison of experiences between those living on and off base during their partner’s deployment. Moreover, the participants who willingly participated in this study could be qualitatively different from individuals who chose not to participate. For example, the participants in the present study could have been less stressed than their peers (i.e., other at-home partners) experiencing military deployments and therefore willing to commit to the responsibilities associated with a three-week-long study. In future investigations, researchers should include more control variables, such as participants’ social support networks, pre-existing mental and physical health conditions, and socioeconomic statuses. For example, at-home partners without financial stress and with good occupations and family support may feel more secure during deployments and in general. In the present study, we checked for significant differences between college graduates and non-college graduates on all our variables of interest and there were none. However, it is possible that college graduates have more sophisticated coping strategies at their disposal during deployments, so educational level should be considered in future studies.

Future research should also measure stress in alternative ways to self-reports, such as physiological or behavioral assessments, as well as consider how PTSD associates with relationship satisfaction and whether writing interventions can help service members, their partners, and their families cope with PTSD more effectively. This is important to consider given research showing that PTSD has many negative effects on military couples and families [74]. Finally, although our sample for the experiment was sufficient for detecting findings with moderate effect sizes, a larger sample size would be needed to detect findings with smaller effects.

Another limitation is that data were only collected at two time points. Obtaining a more precise picture of attachment during the deployment process would include more time points, including before, during, and after deployment [9]. This type of longitudinal design, which would include gathering baseline data before deployment, would allow for causal claims and enable researchers to determine the extent to which the attachment system was activated based on deployment characteristics and one’s attachment styles. The period immediately following deployment, when partners are reunited, is another important data point to consider for future research. In the present study, there was no difference in perceptions of deployment stress based on whether one’s partner was still deployed or had recently returned. The adjustment period following deployment may be stressful and fraught with uncertainty for some couples as they reintegrate their routines and renegotiate boundaries related to connection and autonomy [30]. Longitudinal studies could also better determine how the degree of time spent engaged in affectionate writing and the intervals between writing sessions impact changes in attachment. Recent work indicates that optimal results are most likely when expressive writing sections are spaced close together within a week, suggesting that this study’s findings may have been stronger if such instructions had been given [49]. Qualitative assessments of the content of expressive writing would also be helpful to determine if specific properties of the written text associate with attachment style. Such research would also give scholars a deeper understanding of the lived experiences of participants during the deployment process.

Dyadic data could build on the present study’s findings by ascertaining the roles that both the service person’s and the stay-at-home partner’s attachment styles play during the deployment process. This study focused on the stay-at-home partner, but the service member is also dealing with separation. Understanding how attachment styles and affectionate writing influence the service member’s experiences is critical, as well as understanding how partners’ experiences intersect. For couples with children, it is also especially important to look at deployment stress within the context of the broader family structure [74].

Despite these limitations, the current study extends research and theory on attachment during separation by looking at this issue within the context of military couples experiencing deployment. Attachment is related to satisfaction and stress, with individuals who have anxious attachment styles (preoccupied and fearful) experiencing less satisfaction and more stress during deployments. The good news is that affectionate writing appears to provide a useful way to combat attachment anxiety during military deployments, with both preoccupied and fearful attachment decreasing significantly when participants completed an affectionate writing task. This demonstrates the power of the expressive writing paradigm. Interventions involving affectionate writing may also complement other interventions that promote anticipated relational savoring [4], encourage emotional expression [43,66], and foster connection [41]. Such activities function to keep an attachment bond strong despite physical separation.

New studies should also determine how attachment functions in other situations where separation can trigger the attachment system. For example, couples in long-distance relationships or couples that include a partner who travels a lot for work may also benefit from engaging in affectionate writing, perhaps through journaling. Such work would help scholars to further understand how people can cope with separation in ways that foster attachment security and healthy relationships.

## Figures and Tables

**Table 1:** Analysis of variance results testing group equivalency on selected variables by condition.

Dependent Variable	Affectionate Writing	Innocuous Writing	No Writing	*F*-Value
Participant Age	29.12 (5.98)	31.96 (6.54)	28.93 (5.97)	2.04
Years in the Military	3.94 (1.52)	4.36 (1.95)	4.72 (1.90)	0.43
Weeks Deployed	14.88 (4.24)	14.81 (6.26)	14.79 (6.21)	1.30
Previous Times Deployed	3.28 (1.81)	3.26 (1.51)	3.54(1.75)	0.23
Relationship Length (in years)	6.04 (3.92)	8.04 (5.02)	5.96 (4.24)	0.81
Secure Attachment (Time 1)	5.59 (0.57)	5.75 (0.72)	5.62 (0.80)	0.38
Preoccupied Attachment (Time 1)	4.77 (0.99)	5.07 (1.11)	4.68 (1.04)	1.01
Fearful Attachment (Time 1)	4.67 (1.21)	4.90 (1.38)	4.47 (1.33)	2.10
Dismissive Attachment (Time 1)	5.13 (1.10)	5.67 (0.91)	5.20 (1.09)	0.76

Notes. All results were non-significant at *p* < 0.05. Standard deviations are shown in parentheses. *n* = 80.

**Table 2:** *t*-test results comparing those whose partners returned versus were still deployed by the end of this study on Time 2 variables.

Dependent Variable	Returned	Deployed	*t*-Value
Relational Satisfaction	5.66 (0.76)	5.81 (0.80)	−0.86
Deployment Stress	2.91 (0.42)	2.83 (0.49)	1.23
Secure Attachment	5.68 (0.81)	5.68 (0.81)	−0.10
Preoccupied Attachment	4.58 (1.24)	4.50 (1.06)	0.30
Fearful Attachment	4.22 (1.61)	4.13 (1.48)	0.26
Dismissive Attachment	5.27 (0.93)	5.43 (0.81)	−0.77

Notes. All results were non-significant at *p* < 0.05. Standard deviations are shown in parentheses. *n* = 80.

**Table 3:** Results for models regressing relational satisfaction and deployment stress on attachment styles.

Criterion Variable	Predictors	b	SE	β	*t*
Relational Satisfaction	Secure Attachment	0.58	0.08	0.68	7.54 ***
	Preoccupied Attachment	−0.23	0.07	−0.41	−3.41 **
	Dismissive Attachment	0.05	0.06	0.09	0.92
	Fearful Attachment	−0.18	0.05	−0.40	−3.40 **
Deployment Stress	Number of Previous Deployments	−0.04	0.03	−0.16	−1.65
	Secure Attachment	−0.15	0.07	−0.22	−2.09 *
	Preoccupied Attachment	0.17	0.06	0.39	2.65 **
	Dismissive Attachment	−0.12	0.05	−0.27	−2.23 *
	Fearful Attachment	0.14	0.05	0.40	2.92 **

Notes. * *p* < 0.05; ** *p* < 0.01; *** *p* < 0.001; *n* = 80.

## Data Availability

The data presented in this study are available on request from the authors.

## Authors’ Note

This research was conducted when both authors were at Arizona State University. The first author is currently a social fitness scientist with the Henry M. Jackson Foundation. The opinions and assertions expressed herein are those of the authors and do not reflect the official policy or position of the Uniformed Services University or the Department of Defense. The contents of this publication are the sole responsibility of the authors and do not necessarily reflect the views, opinions, or policies of The Henry M. Jackson Foundation for the Advancement of Military Medicine, Inc. Mention of trade names, commercial products, or organizations does not imply endorsement by the U.S. Government.
